# Maternal effects and the outcome of interspecific competition

**DOI:** 10.1002/ece3.7586

**Published:** 2021-05-02

**Authors:** Benjamin Van Allen, Natalie Jones, Benjamin Gilbert, Kelly Carscadden, Rachel Germain

**Affiliations:** ^1^ Ecology, Behavior, and Evolution University of California San Diego San Diego CA USA; ^2^ School of Biological Sciences University of Queensland Brisbane Qld Australia; ^3^ Department of Ecology and Evolutionary Biology University of Toronto Toronto ON Canada; ^4^ Ecology and Evolutionary Biology University of Colorado Boulder Boulder CO USA; ^5^ Zoology & Biodiversity Research Centre The University of British Columbia Vancouver BC Canada

**Keywords:** competitive asymmetry, environmental quality, lag effects, niche partitioning, temporal autocorrelation, time series analysis

## Abstract

1. Maternal environmental effects create lagged population responses to past environments. Although they are ubiquitous and vary in expression across taxa, it remains unclear if and how their presence alters competitive interactions in ecological communities.
2. Here, we use a discrete‐time competition model to simulate how maternal effects alter competitive dynamics in fluctuating and constant environments. Further, we explore how omitting maternal effects alter estimates of known model parameters from observational time series data.
3. Our simulations demonstrate that (i) maternal effects change competitive outcomes, regardless of whether competitors otherwise interact neutrally or exhibit non‐neutral competitive differences, (ii) the consequences of maternal effects for competitive outcomes are mediated by the temporal structure of environmental variation, (iii) even in constant conditions, competitive outcomes are influenced by species' maternal effects strategies, and (iv) in observational time series data, omitting maternal effects reduces variation explained by models and biases parameter estimates, including competition coefficients.
4. Our findings demonstrate that the ecological consequences of maternal effects hinge on the competitive environment. Evolutionary biologists have long recognized that maternal effects can be an important but often overlooked strategy buffering populations from environmental change. We suggest that maternal effects are similarly critical to ecology and call for research into maternal effects as drivers of dynamics in populations and communities.

## Graphical abstract

Maternal effects can overturn competitive differences among species, driving patterns of coexistence or exclusion that would not be possible in their absence. Who wins and who loses depend on the particular strategies pairs of species employ, as well as how variable the environment they are found in is. As such, maternal effects may act as a species' niche dimension.

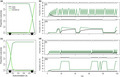

## 1 INTRODUCTION

A central goal of community ecology is to determine how the biotic and abiotic environment interact to maintain diverse species assemblages (Chase & Leibold, 2003; Chesson, 2000; Leibold & Chase, 2017), and the role temporal environmental variability plays in biodiversity maintenance is increasingly recognized (Adler et al., 2010). In order for temporally variable conditions to maintain diversity, species must either differ in which environments are most favorable or differ in their sensitivities to environmental variation (Yuan & Chesson, 2015). As a result, species with identical resource requirements may co‐occur even if they could not stably coexist in constant conditions because they differ in when their populations most strongly impact those resources (e.g., warm vs. cool years; Tilman et al., 1981).

Models of temporal coexistence usually assume that per capita demographic rates at one time point are independent of conditions at a previous point in time (Adler et al., 2006; Levine & Rees, 2004); however, this assumption is broken for species that exhibit “parental environmental effects.” Parental environmental effects are present when an individual's phenotype is determined not only by the environment it experiences, but also by the environment experienced by its parents, most often its mother (henceforth “maternal effects” for brevity; Moore et al., 2019; Mousseau & Fox, 1998; Roach & Wulff, 1987). When these phenotypic effects translate into altered demographic rates, maternal effects introduce lagged responses to population dynamics. For example, maternal effects can alter population growth rates at low densities (Germain & Gilbert, 2014), changing the relative performance of competing species through time, potentially strongly enough to alter the outcome of competition. Theory integrating maternal effects is central to improving predictions of coexistence in variable environments, as well as to clarify the ecological importance of maternal effects more generally.

The long‐term ecological consequences of maternal effects should depend on the structure of temporal environmental variation. Environments vary in how “autocorrelated” they are in space and time (Vasseur & Yodzis, 2004), where environments with high temporal autocorrelation have similar conditions over long timescales. Even in the absence of maternal effects, temporal autocorrelation can structure community diversity, for example, if environmental fluctuations allow recovery and persistence of rare species (Burgess & Marshall, 2014; Levine & Rees, 2004) or change extinction risks (Adler & Drake, 2008). Maternal effects are commonly assumed to confer a fitness advantage to offspring when the offspring environment is highly similar to the maternal environment (temporally autocorrelated) or can be anticipated by mothers (i.e., also referred to as “anticipatory maternal effects” [Marshall & Uller, 2007]). Yet, it is not known how maternal effects interact with environmental autocorrelation (a knowledge gap also highlighted in Burgess & Marshall, 2014) and whether the impacts of maternal effects on performance are maintained in the presence of competitors.

**FIGURE 1:**
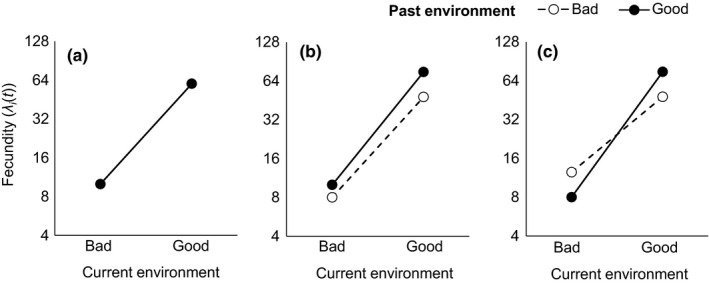
Reaction norms depicting a focal species which exhibits (a) no maternal effect (i.e., past environment has no effect), (b) a silver spoon maternal effect, or (c) environmental matching maternal effect, in two types of offspring environments: bad versus good. Solid circles and solid lines show individuals whose mothers experienced a good environment, while open circles and dashed lines show individuals whose mothers experienced a bad environment. *M* can differ in value depending on conditions in the maternal (m) and offspring (o) generation: *M*_m,o_. In (b), *M*_gb_ and *M*_gg_ are 1, whereas *M*_bg_ and *M*_bb_ are −1, and in (c), *M*_gg_ and *M*_bb_ are 1, whereas *M*_bg_ and *M*_gb_ are −1. As such, using Equation (2), (a) *λ_i_*(*t*) is 10 or 60 in a bad or good current environment, respectively, when no maternal effects are present. (b) When a species exhibits a silver spoon phenotype, *λ_i_*(*t*) is 8 in bad current conditions with a bad past, 10.25 in bad current conditions with a good past, 48 in good current conditions with a bad past, and 75 in good current conditions with a good past. (c) When a species exhibits an environmental matching phenotype, *λ_i_*(*t*) is 10.25 in bad current conditions with a bad past, 8 in bad current conditions with a good past, 48 in good current conditions with a bad past, and 75 in good current conditions with a good past. Note the *y* axis is on a log_2_ scale to aid visualization; see Table S1 for additional details

Not all maternal effects are the same, and different “forms” of maternal effects might alter the outcome of competition in different ways. Here, we use reaction norm plots to visualize different forms of maternal effects, showing how offspring fitness depends jointly on maternal and offspring environmental conditions (Figure 1; Monaghan, 2008; Stearns, 1992). For example, two forms of maternal effects that lead to different reaction norms of offspring fitness across maternal and offspring environmental conditions are “silver spoon” and “environmental matching” (Figure 1; Grafen, 1988; Monaghan, 2008). A silver spoon phenotype occurs when mothers that experienced good conditions produce offspring with higher relative fitness in any offspring environment (Figure 1b)—this type of maternal effect is generally underlain by overall increases in offspring resource provisioning when the maternal environment is favorable, as well as by other mechanisms. This life history response is seen across taxa in plants, invertebrates, birds, and mammals (Harrison et al., 2011; Rossiter, 1996; Sultan et al., 2009; Van Allen & Rudolf, 2013; Van de Pol et al., 2006). By contrast, an environmental matching phenotype (a form of anticipatory maternal effects; Marshall & Uller, 2007) is a life history where offspring perform better when their environment matches their mother's environment. Thus, with an environmental matching phenotype, comparing offspring fitness from different good and bad maternal environments yields a characteristic crossing of reaction norms across offspring environments (Figure 1c). This pattern is thought to arise due to the costs of the maternal environment poorly predicting the offspring environment, for example, if traits adaptive in bad environments (e.g., conserving resources) are maladaptive in good environments, and vice versa (Bateson et al., 2004; Burgess & Marshall, 2014; Galloway & Etterson, 2007; Sultan et al., 2009).

Silver spoon and environmental matching maternal effects are common and can differ in adaptive value depending on ecological context (Germain & Gilbert, 2014; Herman & Sultan, 2011; Marshall et al., 2010; Marshall & Uller, 2007; Mousseau & Fox, 1998; Roach & Wulff, 1987; Sultan et al., 2009; Van Allen & Rudolf, 2015). For example, Sultan et al. (2009) found that *Polygonum* congeners responded differently to maternal and offspring soil moisture conditions. The more generalist species exhibited an environmental matching response, which was adaptive in consecutive dry years. By contrast, the wetland specialist exhibited a silver spoon response that would privilege offspring from wet maternal environments, but in consecutive dry years, this strategy was maladaptive. The consequences of environmental matching in a variable world could have great implications for species distributions as climate change reduces autocorrelation among years, thus reducing fitness (Jacob et al., 2015; Marshall & Burgess, 2015; Marshall et al., 2010). However, while studies have found that maternal effects can alter competitive interactions (Agrawal, 2001; Allen & Marshall, 2010; Duckworth et al., 2015; Fox et al., 1999; Jacob et al., 2015; Mousseau & Dingle, 1991; Stratton, 1989; Van Allen & Rudolf, 2016), no study to date has examined whether the advantage of a given form of maternal effect holds in the presence of interspecific competitors, where outcomes depend not on absolute performance of offspring in different environments, but rather on performance relative to interspecific competitors.

We use a deterministic discrete‐time Beverton–Holt model to explore the influence of maternal effects on competitive interactions and mechanisms of coexistence through time among competing species. We examine scenarios in which neither, one, or both competing species exhibit either a silver spoon or environmental matching life history strategy, modeled as changes to offspring fecundity in response to environmental quality (“good” vs. “bad”). For illustrative purposes, we frame our simulations in terms of annual plant communities, although our models are general to organisms with discrete life cycles and nonoverlapping generations. We use the model to answer four questions: (1) How do maternal effects alter population dynamics and species coexistence? (2) How do their effects vary under different scenarios of temporal autocorrelation? (3) How do their effects interact with existing competitive differences among species? (4) How does the presence of unmeasured maternal effects bias population parameter estimation from time series data? We predict that different forms of maternal effects are optimal under different levels of temporal autocorrelation, leading to scenarios where the addition of maternal effects to ecological dynamics could have strong impacts on competitive interactions and outcomes. Specifically, we predict that species with environmental matching phenotypes benefit from increased autocorrelation, whereas species with a silver spoon phenotype benefit as good years increase in frequency. Our aim is to clarify how variation in temporal autocorrelation can lead to turnover in competitive hierarchies when species with different maternal effects phenotypes compete.

## 2 MODELING POPULATION CONSEQUENCES OF MATERNAL EFFECTS

### 2.1 Setting up the model

We begin investigating the impact maternal effects have on population dynamics by modifying a discrete‐time Beverton–Holt annual plant model (Levine & HilleRisLambers, 2009) that describes the dynamics of competing annual plant populations with the influence of maternal effects:

(1) $$N_{i}\left(t+1\right)=\frac{N_{i}\left(t\right)\lambda_{i}\left(t\right)}{1+\alpha_{\mathrm{ii}}N_{i}\left(t\right)+\alpha_{\mathrm{ij}}N_{j}\left(t\right)},$$

where *N_i_*(*t*) is the population size of species *i* at time *t*, thus, the population is increasing in size when *N_i_*(*t* + 1) > *N_i_*(*t*). The intrinsic rate of increase for species *i* is *λ_i_*(*t*)—we refer to this term as the “fecundity” parameter, which we can modify to introduce maternal environmental effects. Specifically, as we will describe, the value of *λ_i_*(*t*) can vary at any given time *t* depending on current conditions (i.e., the offspring environment) and conditions at *t* − 1 (i.e., the maternal environment). The competitive effect species *i* has on itself is given by *α_ii_*, whereas the interspecific effect of species *j* on species *i* is *α_ij_*. The value of *α_ii_* is assumed to be 1 for simplicity; thus, the strength of *α_ij_* can easily be compared to *α_ii_*. Because Equation 1 is symmetric, the population dynamics of species *j* are modeled by switching subscripts *i* and *j*. We note that Equation (1) can be modified to accommodate a persistent seed bank; however for simplicity, we assume no seed bank, and as a result, Equation 1 tracks the number of germinated plants as population size.

We used Equation (2) to modify *λ_i_* at each timepoint *t* based on the quality of conditions in the maternal (i.e., time *t* − 1, denoted by subscript m) and offspring (i.e., time *t*, denoted by subscript o) generation:

(2) $$\lambda_{i}\left(t\right)=\lambda_{\left(i,o\right)}s_{m}^{M}.$$

Here, term *λ*_(_ *_i_*_,o)_ is the intrinsic rate of increase of species *i* in a given offspring environment in the *absence* of maternal environmental effects, aka prior to any modification. For simplicity, and to follow previous work on the individual and population‐level impacts of maternal effects, the environment in any given year was modeled in binary terms (“good,” i.e., abundant food, wet, etc. vs. “bad,” i.e., scarce food, dry, etc.; Bateson et al., 2004; Germain & Gilbert, 2014; McGinley et al., 1987; Monaghan, 2008; Smith & Fretwell, 1974; Van Allen & Rudolf, 2013), with “bad” the environment in which a species has a lower *λ*_(_ *_i_*_,o)_. We modeled maternal effects that manifest through offspring traits (*s*) based on maternal conditions m (again, good vs. bad). When *s* = 1, offspring traits are unaffected by conditions in the maternal generation, meaning that fecundity at any given time step *t* is purely determined by whether or not offspring are in a good or bad environment. When *s* ≠ 1, offspring traits are affected by conditions in the maternal generation, agnostic to whether the offspring environment is good or bad. As we described, each form of maternal effect (i.e., silver spoon vs. adaptive matching) differ in how phenotypic changes due to maternal conditions translate into increased or decreased fecundity—this translation is described by parameter *M*. Specifically, because *s*_m_ is raised to the power of *M*, maternal effects on phenotypic traits do not modify *λ*_(_ *_i_*_,o)_ when *M* equals 0 (i.e., $S_{m}^{0}=1$, thus $\lambda_{\left(i,o\right)}\times s_{m}^{0}=\lambda_{\left(i,o\right)}$). When *M* equals 1, *λ*_(_ *_i_*_,o)_ is modified proportionally to *s*_m_, such that a 20% increase in a phenotypic trait leads to a 20% increase in *λ*_(_ *_i_*_,o)_, and the inverse is true when *M* equals −1. As we will discuss, parameter *M* is species‐specific and generalizes the model to include different forms of maternal effects (e.g., environmental matching; Figure 1; Table S1).

For our analyses, we make three simplifying assumptions. First, we assume that maternal effects are based solely on environmental conditions in the previous year rather than accumulating over time (i.e., no grandparental effects, but see Bateson et al., 2004; Beckerman et al., 2003; Herman et al., 2012). Second, we assume that maternal effects have a moderate effect on phenotypic traits that, depending on the value of *M*, directly translate into a proportional change in fecundity, on the order of a 20% reduction or enhancement compared to offspring that do not exhibit maternal effects—in other words, *s* was set to either 0.8 or 0.8^−1^ depending on maternal conditions. This effect size is arbitrary but falls within the natural range of effects on fecundity observed in plants and animals (e.g., Fox et al., 1997; Galloway, 2005; Moore et al., 2019), and any other magnitude of proportional change would simply act to magnify or dampen any influences of maternal effects we observe. By modeling maternal effects through phenotypic traits which affect fecundity, without focusing on any specific trait in particular, our approach is general and agnostic to exact biological mechanisms. In plants, examples of maternal effects that impact fitness include changes to seed size or quality, plant root or shoot growth, and anti‐herbivore defenses (Galloway & Etterson, 2007; Germain et al., 2013; Herman & Sultan, 2011; Jakobsson & Eriksson, 2000; Roach & Wulff, 1987; Smith & Fretwell, 1974; Vance, 1973). Third, we constrain our analyses to the impacts of maternal effects on competitive outcomes and omit the reciprocal impact that competition may have on the evolution of maternal effects, for example, through seed provisioning (Geritz, 1995; Rees & Westoby, 1997).

### 2.2 Simulating different ecological scenarios

We contrast the consequences of maternal effects on the competitive dynamics of two types of species pairs: species that are competitively equivalent and are thus interacting neutrally, and species that interact non‐neutrally, exhibiting a clear competitive hierarchy with some level of niche partitioning (i.e., “stabilizing” coexistence of unequal competitors, in which intraspecific competition > interspecific competition; Chesson, 2000). In both scenarios, species have identical intrinsic rates of increase, which are 6× greater when offspring conditions are good vs. bad; *λ*_(_ *_i_*_,o=“good”)_ = 60, *λ*_(_ *_i_*_,o=“bad”)_ = 10). As stated above, *λ_i_*(*t*) is modified depending on conditions experienced in the past and current environment via Equation (2), as described in Figure 1 and Table S1. Neutrality is the case where intraspecific and interspecific competition is equivalent, here modeled as *α_ii_* = *α_jj_* = *α_ij_* = *α_ji_* = 1.0; stable coexistence is impossible among neutrally interacting species; thus, our model examines if and when adding maternal effects alter competitive outcomes. By contrast, the non‐neutral species pair takes on parameter values *α_ii_* = 1.25, *α_jj_* = 1.0, *α_ij_* = 1.0, and *α_ji_* = 0.8. These specific parameter values, when intraspecific competition exceeds interspecific competition, allow each species to increase from low abundance, thus stabilizing coexistence. However, these values also allow a competitive hierarchy because species *j* is less sensitive to competition than species *i* and is thus a superior competitor. We can explore when maternal effects reinforce or counteract this existing competitive hierarchy to decrease or increase the possibility of coexistence via stabilization. Although we present only two sets of parameters for simplicity here, we discuss their generality in the Section 4 and in Figure S1.

Using the population model in Equation (1), we simulated the population dynamics of competitors under varying scenarios of interannual variation in environmental quality and forms of maternal effects using parameter sets described above. Each simulation ran for 100 time steps (i.e., years/generations for annual plants), with good and bad environments drawn with equal probability. Two individuals of each species were added at time *t* = 2, which allowed a lagged response of environmental conditions at the start of the model run in *t* = 1. We explore different scenarios of temporal autocorrelation (*k*), from 0 to 1 in 0.1 increments; 0.5 autocorrelation means that the environment is equally likely to switch or remain constant among *t* − 1 and *t*; 0 means that the environment always switches each time step, and 1 means that the environment remains constant. Even though the probabilities of sampling good versus bad environments and of switching are user‐specified, they are still probabilities, meaning that random sampling causes observed proportions of good versus bad years and switching versus no switching to deviate from those expected. As such, we reran each model 500 times to gain a more accurate picture of central tendency and range of outcomes.

### 2.3 Estimating maternal effects from simulated time series data

To quantify how omitting maternal effects from population growth models can bias population parameters estimated from time series data, we used Equation 1 to simulate the dynamics of an empirically parameterized pair of species (*Vulpia microstachys* and *V*. *octoflora*; parameter estimates from (Germain et al., 2016); values shown in Table 1) under different maternal effects scenarios. We ran the simulation for 500 years with an intermediate level of temporal autocorrelation (*k* = 0.5) under three competitive scenarios: (i) neither species exhibits a maternal effect (to confirm that our model fitting procedure was successful); (ii) *V*. *octoflora* exhibits a silver spoon phenotype; and (iii) *V*. *octoflora* exhibits environmental matching. Each competitive scenario was replicated 100 times.

**TABLE 1:** The influence of maternal effects on population parameter estimation when maternal effects are not explicitly estimated

Focal species	Parameter	Actual values	Estimated values		
			Both species	Species *j*	Species *j*
			None	Silver spoon	Matching
Species *i* (*Vulpia microstachys*)	*λ_i_*(*g*)	236	236.0	236.0	236.0
	*λ_i_*(*b*)	153	152.4	152.4	152.4
	*α_ii_*(*g*)	0.099	0.099	0.099	0.099
	*α_ii_*(*b*)	0.082	0.082	0.082	0.082
	*α_ij_*(*g*)	0.083	0.083	0.083	0.083
	*α_ij_*(*b*)	0.002	0.002	0.002	0.002
	Model *R* ^2^	—	1.0	1.0	1.0
Species *j* (*Vulpia octoflora*)	*λ_j_*(*g*)	924	923.0	**787.4**	**865.0**
	*λ_j_*(*b*)	1,127	1,126.9	**1,027.1**	**1947.0**
	*α_jj_*(*g*)	0.456	0.455	**0.296**	**0.296**
	*α_jj_*(*b*)	0.515	0.514	**0.345**	**1.353**
	*α_ji_*(*g*)	0.302	0.302	**0.270**	**0.301**
	*α_ji_*(*b*)	0.574	0.575	**0.531**	**0.860**
	Model *R* ^2^	—	1.0	0.87	0.83

Note

The purpose of the “none” scenario was to confirm that model fitting was successful. For brevity, we only modeled scenarios in which species *j* exhibited one of three phenotypes, competed against species *i* with no maternal effects. Each parameter was parameterized in a good (i.e., adequate water) and bad (i.e., restricted water) environmental condition.

Bold text indicates estimates which differ from the actual values.

For each of the three scenarios above, we used nonlinear least squares regression (R package “nls”) to fit Equation 1 to the simulated time series data and estimate each parameter in Equation 1 assuming no maternal effects (i.e., *λ_i_* = *λ_i_*_,o_). We allowed the model to fit separate estimates of *λ* and *α* in good or bad offspring environments by including environmental quality as a dummy variable for each parameter, as the empirical data were used to generate the time series data were parameterized in two environments which differed in quality (i.e., wet vs. dry; Germain et al., 2016). We use these data to test how closely the fitted parameter estimates resemble the actual values used to generate the time series data, and how much variation in population dynamics is unexplained when lagged responses to maternal environmental conditions are not considered (i.e., by comparing *R* ^2^). Although one might intuitively expect the model omitting maternal effects to fit the simulated data less strongly than a model that was used to simulate the data in the first place, here we are most interested in (i) the magnitude of this difference; (ii) how estimates of specific demographic parameters are affected and in what direction; and (iii) which form of maternal effects (i.e., silver spoon vs. environmental matching) alters the outcome the most.

## 3 RESULTS

**FIGURE 2:**
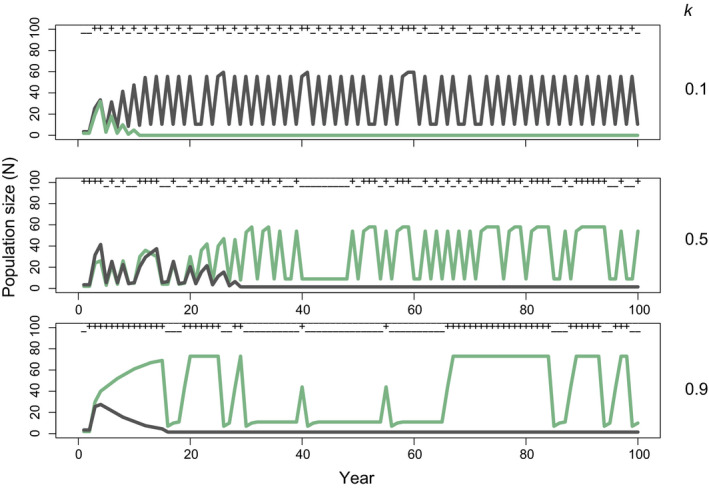
Competitive dynamics of a species with no maternal effect (gray lines) versus a species that exhibits environmental matching (dark green lines) at (a) low, (b) intermediate, and (c) high levels of temporal autocorrelation (*k*). Environmental conditions in each year, good (+) and bad (−), are shown at top of each panel, and only the first 100 years of each simulation are shown. These simulations correspond to Figure 3c, with levels of autocorrelation shown indicated by the stars; similar plots for other competitor combinations are presented in the Supporting Information. Note that each replicate simulation produces a different environmental structure

**FIGURE 3:**
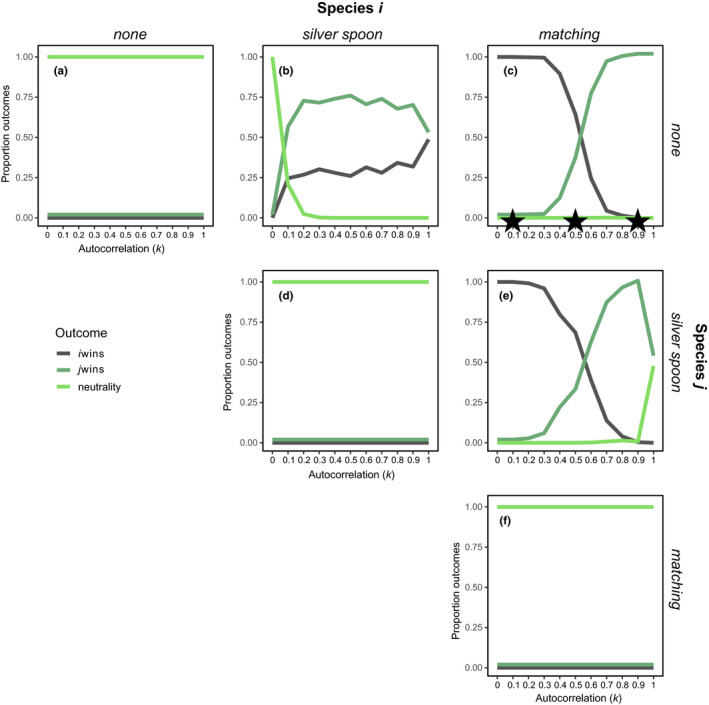
Proportion of simulation outcomes in which only species *i* (gray line), only species *j* (dark green line), or both species (bright green line) are present at the end of the 500‐year simulation, depending on the type of maternal effects exhibited by each species (different combinations shown in each of panels (a‐f)) and the level of temporal autocorrelation (*k*). Autocorrelation of 1.0 corresponds to constant conditions, 0.5 is a 50% chance of switching conditions among years, and 0.0 is 100% chance of switching (see Figure 2, e.g., of how changing k affects the temporal sequence of environmental conditions). Because species *i* and *j* are competitively equivalent in all ways except for maternal environmental effects, stable coexistence is impossible; thus, we refer to the long‐term maintenance of both species in these simulations as “neutrality” as any demographic stochasticity could produce random extinctions. The stars in (c) correspond to the three scenarios shown in Figure 2. Competition coefficients are *α_ii_* = *α_jj_* = *α_ij_* = *α_ji_* = 1.0

Our simulations demonstrate that, for any given species, persistence depends on the three‐way interaction between (i) their own maternal effect strategy; (ii) the maternal effect strategy expressed by their competitor; and (iii) the structure of temporal autocorrelation (Figures 2 and 3). When environmental conditions are almost always constant from year to year (high autocorrelation), species possessing an environmental matching phenotype usually perform best. However, there is one exception: Under constantly good conditions, which probabilistically arise in 50% of replicate simulations when *k* = 1, species possessing either an environmental matching or a silver spoon phenotype perform equivalently (since their fitness in optimal conditions is set to be equivalent in our model), but for different reasons. Any amount of switching among environments above *k* = 0.5, even under high autocorrelation (e.g., *k* = 0.9 in Figure 3e), gives the environmental matching species an advantage over the silver spoon species. When autocorrelation is low, the environmental matching species is at a severe disadvantage, as frequent switching among environments means that this species has depressed population growth (*M* of −1) most of the time (Figures 2a and 3c,e). The transition point at which an environmental matching phenotype performs better or worse than competitors occurs at an autocorrelation of 0.5 (Figures 2b and 3c,e)—that is, where the probability of the environment switching is equal to the probability of the environment remaining constant.

When a species with a silver spoon phenotype competes with a species with no maternal effect, the probability of either species winning varies nonlinearly with temporal autocorrelation (Figure 3b). In the absence of autocorrelation (*k* = 0), both species compete equivalently, because geometric averaging with fluctuations among good and bad years equalizes their long‐term population growth rates. When environments are perfectly autocorrelated (*k* = 1), the silver spoon species dominates in 50% of simulations (when the environment is constantly good), whereas the species with no maternal effect dominates in the other 50% of the simulations, when conditions are constantly bad. At low to intermediate levels of autocorrelation (0.1 > *k* > 0.6), the species with no maternal effect dominates in the majority (>50%) of the simulations, whereas at intermediate to high levels of autocorrelation (0.6 > *k* > 0.9), the two types of species trend toward dominating in an equal number of simulations. As expected, when neutrally interacting species express identical maternal effects, competitive equivalence is maintained, and thus, maternal effects do not influence the outcome of competition (Figure 3a,d,f). These results highlight the complex influence maternal effects have on competitive outcomes, even in a simple model with no other form of competitive differentiation.

When we examine the population dynamics among competitors in our simulations (Figure 2), the influence of temporal environmental structure becomes clear. For example, examining dynamics between a species that does not express a maternal effect (gray line in Figure 2) and a competitor that expresses an environmental matching phenotype (green line in Figure 2), we see clear switching in the identity of the winner as the environment becomes increasingly autocorrelated. What is evident from these simulations is that both species can transiently persist, and the duration of persistence depends on the exact sequence of environments that are sampled through time. We can see in Figure 3 that, across replicated simulations, both species win in equal frequency at *k* = 0.5. In this particular replicate, we see the loss of the species that lacks a maternal effect as the proportion of constant years exceeds the proportion of transition years for a long enough interval (between years 15 and 30). In other replicates, transition years are more frequent, instead causing the loss of the environmental matching species. Although we show this specific competitive pair as an example here, the dynamics of the others are summarized in Figure 3 and shown in Figure S2.

**FIGURE 4:**
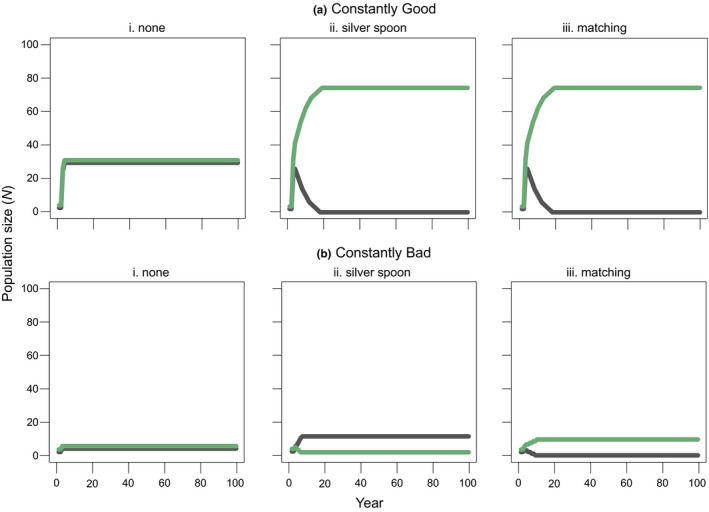
The influence of one species exhibiting a maternal environmental effect in (a) constantly good and (b) constantly bad conditions (i.e., *k* = 1.0). Species *i* (gray lines) has no maternal effect, whereas species *j* (dark green lines) exhibits the type of maternal effect indicated in the panel headers. Panels (i) represent dynamics in the absence of maternal environmental effects, where both species neutrally persist in the absence of demographic stochasticity. Panels (ii) represent dynamics when silver spoon maternal effects occur for species *j* and panels (iii) represent dynamics when matching maternal effects occur for species *j*

Maternal effects influence competitive outcomes even in constant conditions. We specifically examine cases where a species with no maternal effect (gray lines in Figure 4) competes with a species with either a silver spoon (green lines in Figure 4a[ii],b[ii]) or environmental matching phenotype (green lines in Figure 4a[iii],b[iii]). The “none” scenario shows a case where neither competitor exhibits a maternal effect and demonstrates how outcomes are identical regardless of environment type (Figure 4a[i],b[i]). By contrast, the species that outcompetes the other (Figure 4a[ii],b[ii]) or attains the greatest population size (Figure 4a[iii],b[iii]) with maternal effects differs from in the “none” scenario. The species with a silver spoon phenotype competitively excludes the species that lacks a maternal effect in constantly good conditions but is excluded by it in constantly bad conditions—these outcomes are predicted from their relative differences in fecundity among species in each environment type (Figure 1b vs. Figure 1c).

**FIGURE 5:**
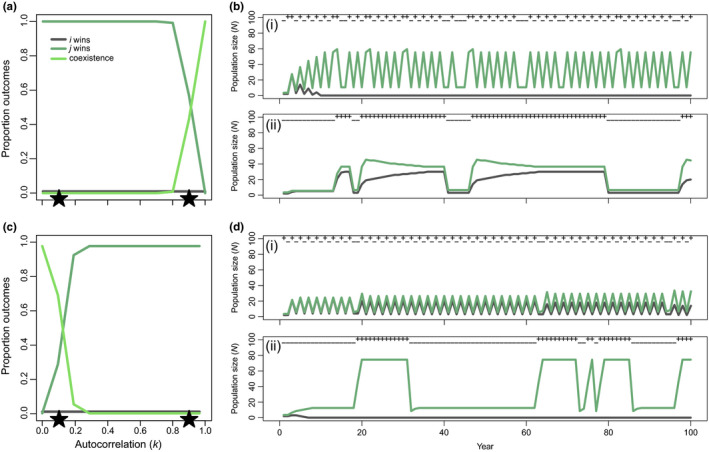
Maternal effects can alter competitive outcomes in a scenario in which species *j* (dark green lines) is competitively superior to species *i* (dark gray lines), and an environmental matching phenotype is either expressed (a, b) by species *i* or (c, d) by species *j*. In the absence of maternal effects, species *j* would exclude species *i* in all simulations. Dynamics shown in panels (b) and (d) correspond to the stars in panels (a) and (c), respectively, and are meant to show the temporal dynamics in more detail. Only two competitive pairs are shown for brevity, but all pairwise combinations are in Figure S3. Competition coefficients are *α_ii_* = 1.25, *α_jj_* = 1.0, *α_ij_* = 1.0, and *α_ji_* = 0.8

By introducing maternal effects to the dynamics of a pair of species that exhibit a clear competitive hierarchy but also experience some level of stabilization, we can isolate the impacts maternal effects can have on competition (Figure 5). In the absence of maternal effects, species *j* excludes species *i* even in the presence of some stabilizing niche differentiation (Figure S3a). However, the presence of maternal effects acts to exacerbate or ameliorate competitive asymmetries among species, with the latter effect permitting coexistence. We see maternal effects permitting coexistence, for example, if the species exhibiting an environmental matching phenotype is the inferior competitor and finds itself in constant conditions (Figure 5b[ii]) or if the environmental matching species is the superior competitor and encounters a frequently switching environment (i.e., a fitness‐reducing environment, Figure 5d[ii]). Note that the influence of maternal effects on competitive dynamics in the non‐neutral simulations is identical to those described for the neutral simulations, except instead of altering which species is excluded by competition, outcomes can now include switching from exclusion to stable coexistence. We present outcomes for all other species pairs in the Supporting Information (Figure S3) but emphasize that the effects we have described can be generalized to any combination of maternal effects that modify fitness (fecundity) inequalities among species under a given level of temporal autocorrelation (Figure S1).

When estimating population parameters from time series data, maternal effects caused estimates to deviate from their actual values and introduced unexplained variation in population dynamics. Specifically, when *V*. *octoflora* exhibited a silver spoon or environmental matching phenotype, all population parameter estimates were affected (bold values in Table 1). The expression of either type of maternal effect resulted in an underestimation of all population parameters in good years—for example, the intraspecific competition coefficient was estimated at 0.296 for both silver spoon and matching phenotypes compared to the actual value of 0.456. In bad conditions, however, the maternal effects phenotypes differed, with silver spoon resulting in an underestimation of all parameters, and matching resulting in an overestimation of all parameters. Not accounting for maternal effects, when present, resulted in a 13%–17% loss in the amount of variation explained by the model, which is otherwise 100% given that our simulations were entirely deterministic. Parameter estimates for the species that did not exhibit a maternal effect were unaffected by the maternal effect exhibited by its competitor.

## 4 DISCUSSION

Maternal effects are well understood to occur across diverse taxa (Bonduriansky & Day, 2009; Chirgwin et al., 2017; Herman & Sultan, 2011; Monaghan, 2008; Moore et al., 2019; Mousseau & Fox, 1998) but their effects on species interactions and coexistence are unresolved. We integrated two common forms of maternal effects into models of population dynamics and coexistence in annual plant communities and found that, even at modest effect sizes, maternal effects altered the strength of, or even reversed, competitive hierarchies and could hasten or prolong competitive exclusion. Maternal effects are often studied in fluctuating environments as they can provide rapid phenotypic change that enhances performance in new conditions (e.g., Badyaev, 2005; Dey et al., 2016; Hoyle & Ezard, 2012; Moore et al., 2019). However, our model highlights that even in stable environments, maternal effects alter the outcome of competition. Below, we discuss general features of our findings, implications for maternal effects studies and ecological experiments, and next steps to exploring the ecological consequences of maternal environmental effects.

We find that maternal effects can influence competitive interactions strongly enough to overturn species' competitive hierarchies. Specifically, the range of dynamics we observe when all else is equal (Figure 3) can be summarized by three rules: (i) a silver spoon species wins against a species with no maternal effect when good years are more frequent than bad years; (ii) an environmental matching species wins against a species with no maternal effect when environmental conditions remain constant more frequently than they switch; and (iii) silver spoon wins over environmental matching when the proportion of good years exceeds the proportion of constant years. Whether or not maternal effects change competitive outcomes (coexistence vs. exclusion) depends on how strongly maternal effects influence relative fitness among competitors compared to other sources of competitive differences. For instance, consider a pair of species with stabilizing niche differences but also dominance of species *j* over species *i* (Figure 5), such that in the absence of maternal effects, species *j* always excludes species *i*. If either species in the pair exhibits an environmental matching phenotype, the outcome of competition depends on the strength of the maternal effect and the level of temporal autocorrelation in the environment. Although this feature might suggest that the influence of maternal effects on ecological dynamics is contingent on pre‐existing competitive differences, it could also be framed that the effects of pre‐existing competitive differences are contingent on the form and strength of maternal effects. The effects we see are general, in that their magnitude or direction of effect on competitive asymmetries did not depend on pre‐existing competitive differences among species, even if outcomes of coexistence or exclusion change (see conceptual figure in Figure S1).

Empirical research finds that an environmental matching phenotype is adaptive in autocorrelated environments with some level of temporal variability, which our results generally support. For example, *Campanula* *americana* has limited seed dispersal, so offspring and maternal plants tend to experience similar environments, and germination is highest in home environments (Galloway, 2005). The same phenomenon has been observed in animal species, such as *Daphnia magna*, which produce offspring with antipredatory helmets in the presence of predators, significantly reducing predation rates (Agrawal et al., 1999). In the absence of predators, the production of helmets is energetically costly, and thus, helmeted offspring are at a fitness disadvantage.

In contrast, silver spoon maternal effects should provide an advantage to individuals reared in a good environment, regardless of the quality or autocorrelation of future environmental conditions. For example, the probability of yellow ground squirrels (*Spermophilus fulvus*) surviving to adulthood increases when they are born in more favorable conditions, such as at low population densities or in more pristine environments (Vasilieva & Tchabovsky, 2020). Similarly, early hatching Crested Ibis (*Nipponia nippon*) enjoy a competitive advantage over later hatching individuals, and this advantage early in life translates to increased adult survival and reproduction as well as the production of larger, healthier offspring (Song et al., 2018). In many species, it is likely that multiple traits could have different maternal effects of different strengths. For example, flour beetles (*Tribolium* sp.) have a matching maternal effect in lifespan, where survival is always reduced when switching from the maternal environment, but a silver spoon effect on fecundity when coming from a good environment always increases it (Van Allen & Rudolf, 2015). For this model, we simplify these dynamics to consider one overall effect on intrinsic growth, in the form of either matching or silver spoon effects.

No experiments have investigated how competitors impact the advantage of environmental matching, where outcomes depend not on absolute performance of offspring in different environments, but rather on performance relative to interspecific competitors. Our simulations suggest that the advantage of environmental matching should generally be maintained in the face of competition. As temporal autocorrelation increases, competitors with environmental matching increasingly dominate against other types of competitors (Figure 3), with one exception. When conditions are constantly good from both competitors point of view, then environmental matching and silver spoon phenotypes both benefit and in our model are then competitively equivalent (Figure 3 at *k* = 1.0). Experiments involving competitors with known differences in maternal effects phenotypes are needed to confirm our theoretical predictions.

Maternal effects are an important component of species' life histories in response to environmental variation, suggesting that maternal effects might contribute to species' geographical distributions (Benard & McCauley, 2008; Duckworth et al., 2015; Marshall & Burgess, 2015; Marshall et al., 2010). As we demonstrate theoretically, in the community context in which we view maternal effects, this could be doubly true, as species that fail to respond optimally to environmental change could be displaced by species that do. As a consequence, maternal effects are likely shaped by selection pressures imposed by environmental variation and community composition (Badyaev, 2005; Mousseau & Fox, 1998; Potticary & Duckworth, 2020; Räsänen & Kruuk, 2007). In turn, the evolution of maternal effects phenotypes determines whether species successfully integrate into an assemblage and establish in different environments (Dey et al., 2016; Duckworth et al., 2015; Dyer et al., 2010; Fox et al., 1999; Mousseau & Fox, 1998; Sultan et al., 2009), for example, species with environmental matching phenotypes occurring more frequently in autocorrelated environments (Marshall & Burgess, 2015). Thus, we hypothesize that the distribution of maternal effects phenotypes in a community matches patterns of variation in habitat quality in that environment. Indeed, studies on regionally co‐occurring plant congeners and insect congeners both found that maternal effects enhance species differences in a way that promotes spatial coexistence, but which tends to reduce local co‐occurrence (Sultan et al., 2009; Van Allen & Rudolf, 2016). In these plant and animal systems, maternal effects are part of what defines each species' niche. If species' distributions across habitat types are linked with their maternal effect strategy, this could, for example, act to reduce invasion by species with the wrong maternal effects phenotype for a given environment or enhance invasion by species with the right maternal effect (Dyer et al., 2010).

In an effort to remove or control for the influences of maternal effects on experimental outcomes, it is common practice to grow all populations and species in a common environment in the generation prior to experimentation (Kawecki & Ebert, 2004). Our model results indicate that even when species are grown under constant conditions for multiple generations, different population growth rates, carrying capacities, and outcomes of competition emerge in constantly good versus constantly bad environments depending on the form of maternal effects competing species express. If maternal effects truly had been removed, population dynamics would be identical in all simulations (Figure 4). Although we show this at the species level, the strength and direction of maternal effects can also vary intraspecifically across genotypes (Germain et al., 2013; Holeski, 2007; Stratton, 1989), so the same dynamics would apply. We suggest that experimental tests more often acknowledge that influences of maternal effects cannot be removed by any experimental design, but rather can be standardized, and that different standardized environments can yield different outcomes; this applies equally to ecological and evolutionary studies. Intriguingly, maternal effects may not only act to obscure or confound signals of adaptation, but actually alter the topography of fitness landscapes, playing a direct role in the evolution of populations and species (Badyaev, 2005; Mousseau & Fox, 1998; Mousseau et al., 2009; Räsänen & Kruuk, 2007).

Observational time series data of species' population growth rates provide invaluable insight into mechanisms that underlie long‐term coexistence (Blüthgen et al., 2016), but might also contain hidden influences of maternal effects. By fitting a standard competition model to abundance data simulated using empirically parameterized population parameters, we found that omitting maternal effects caused population parameter estimates to differ from their known values (Table 1). Surprisingly, this included estimates of competition coefficients, which were not modified by maternal effects in our models. On one hand, our results demonstrate an empirical challenge—how often are population parameter estimates from time series data influenced by maternal effects? On the other hand, this challenge also presents an opportunity—can existing time series data be mined to estimate the presence of time lags imposed by maternal environmental conditions? For example, a recent re‐analysis of multiple published ecological datasets explicitly quantified the strength of time lags (agnostic to the specific mechanism) and found that time lags can account for 15%–28% of unexplained variation (Ogle et al., 2015). Although these time lags were not specific to maternal effects, a similar approach could be applied to existing observational data (Benton et al., 2001; Ginzburg & Taneyhill, 1994).

## 5 CONCLUSION

Much maternal effects research emphasizes the evolutionary ecology of maternal effects—elucidating when they adaptively evolve and how they might buffer populations from rapid environmental change (Badyaev, 2008)—but their importance in ecological communities which include species interactions is less explored (Bernardo, 1996; Moore et al., 2019; Rossiter, 1996; Van Allen & Rudolf, 2016). We show that, in an ecological context, maternal effects can interact with temporal autocorrelation to alter competitive hierarchies and shift coexistence outcomes, even in constant environments. Moreover, maternal effects can act as a hidden source of variation in time series data. Given that exploring the consequences of maternal effects for ecological communities is a new research avenue, we highlight three priority questions to be answered by future studies: (1) What impact do maternal effects have on offspring fitness when they manifest in response to competitors, predators, or other biotic interactors, in addition to the (more commonly studied) abiotic environment (Germain et al., 2018; Larios & Venable, 2015; Tollrian, 1995)? (2) What are the relative impacts of maternal effects in response to spatial heterogeneity or temporal fluctuations in different environments? (3) Can maternal effects be estimated from time series data? If so, how strong are their influences, and how do they differ among species (Ginzburg & Taneyhill, 1994)? Maternal effects are an important dimension of a species’ niche—its ability to persist in its environment, to adapt to it, and, as we show here, its interactions with and effects on the larger biological community.

## CONFLICT OF INTEREST

None declared.

## AUTHOR CONTRIBUTION

**Benjamin Van Allen:** Conceptualization (equal); Data curation (supporting); Formal analysis (equal); Investigation (equal); Methodology (equal); Visualization (supporting); Writing‐original draft (equal); Writing‐review & editing (equal). **Natalie Jones:** Conceptualization (supporting); Formal analysis (supporting); Methodology (supporting); Visualization (supporting); Writing‐review & editing (supporting). **Benjamin Gilbert:** Conceptualization (supporting); Data curation (supporting); Formal analysis (supporting); Funding acquisition (equal); Supervision (supporting); Writing‐review & editing (supporting). **Kelly Carscadden:** Conceptualization (supporting); Formal analysis (supporting); Methodology (supporting); Writing‐original draft (supporting); Writing‐review & editing (supporting). **Rachel Germain:** Conceptualization (equal); Data curation (lead); Formal analysis (lead); Funding acquisition (equal); Writing‐original draft (lead); Writing‐review & editing (equal).

## Supporting information

Supplementary Material

## Data Availability

All codes are publicly available on GitHub, with a doi provided by Zenodo: https://doi.org/10.5281/zenodo.4645299.

## References

[ece37586-bib-0001] Adler, P. B. , & Drake, J. M. (2008). Environmental variation, stochastic extinction, and competitive coexistence. The American Naturalist, 172(5), 186–195. 10.1086/591678 18817458

[ece37586-bib-0002] Adler, P. B. , Ellner, S. P. , & Levine, J. M. (2010). Coexistence of perennial plants: An embarrassment of niches. Ecology Letters, 13, 1019–1029. 10.1111/j.1461-0248.2010.01496.x 20545729

[ece37586-bib-0003] Adler, P. B. , HilleRisLambers, J. , Kyriakidis, P. C. , Guan, Q. , & Levine, J. M. (2006). Climate variability has a stabilizing effect on the coexistence of prairie grasses. Proceedings of the National Academy of Sciences of the United States of America, 103(34), 12793–12798. 10.1073/pnas.0600599103 16908862 PMC1550767

[ece37586-bib-0004] Agrawal, A. A. (2001). Phenotypic plasticity in the interactions and evolution of species. Science, 294(5541), 321–326. 10.1126/science.1060701 11598291

[ece37586-bib-0005] Agrawal, A. A. , Laforsch, C. , & Tollrian, R. (1999). Transgenerational induction of defences in animals and plants. Nature, 401(6748), 60–63.

[ece37586-bib-0006] Allen, R. M. , & Marshall, D. J. (2010). The larval legacy: Cascading effects of recruit phenotype on post‐recruitment interactions. Oikos, 119(12), 1977–1983. 10.1111/j.1600-0706.2010.18682.x

[ece37586-bib-0007] Badyaev, A. V. (2005). Maternal inheritance and rapid evolution of sexual size dimorphism: Passive effects or active strategies? The American Naturalist, 166(Suppl 4), S17–S30. 10.1086/444601 16224709

[ece37586-bib-0008] Badyaev, A. V. (2008). Maternal effects as generators of evolutionary change: A reassessment. Annals of the New York Academy of Sciences, 1133, 151–161. 10.1196/annals.1438.009 18559819

[ece37586-bib-0009] Bateson, P. , Barker, D. , Clutton‐Brock, T. , Deb, D. , D'Udine, B. , Foley, R. A. , Gluckman, P. , Godfrey, K. , Kirkwood, T. , Lahr, M. M. , McNamara, J. , Metcalfe, N. B. , Monaghan, P. , Spencer, H. G. , & Sultan, S. E. (2004). Developmental plasticity and human health. Nature, 430(6998), 419–421. 10.1038/nature02725 15269759

[ece37586-bib-0010] Beckerman, A. P. , Benton, T. G. , Lapsley, C. T. , & Koesters, N. (2003). Talkin' 'bout my generation: Environmental variability and cohort effects. The American Naturalist, 162(6), 754–767. 10.1086/381056 14737713

[ece37586-bib-0011] Benard, M. F. , & McCauley, S. J. (2008). Integrating across life‐history stages: Consequences of natal habitat effects on dispersal. The American Naturalist, 171(5), 553–567. 10.1086/587072 18419566

[ece37586-bib-0012] Benton, T. G. , Ranta, E. , Kaitala, V. , & Beckerman, A. P. (2001). Maternal effects and the stability of population dynamics in noisy environments. The Journal of Animal Ecology, 70(4), 590–599. 10.1046/j.1365-2656.2001.00527.x

[ece37586-bib-0013] Bernardo, J. (1996). Maternal effects in animal ecology. American Zoologist, 36(2), 83–105. 10.1093/icb/36.2.83

[ece37586-bib-0014] Blüthgen, N. , Simons, N. K. , Jung, K. , Prati, D. , Renner, S. C. , Boch, S. , Fischer, M. , Hölzel, N. , Klaus, V. H. , Kleinebecker, T. , Tschapka, M. , Weisser, W. W. , & Gossner, M. M. (2016). Land use imperils plant and animal community stability through changes in asynchrony rather than diversity. Nature Communications, 7, 10697. 10.1038/ncomms10697 PMC4754335 26869180

[ece37586-bib-0015] Bonduriansky, R. , & Day, T. (2009). Nongenetic inheritance and its evolutionary implications. Annual Review of Ecology, Evolution, and Systematics, 40(1), 103–125. 10.1146/annurev.ecolsys.39.110707.173441

[ece37586-bib-0016] Burgess, S. C. , & Marshall, D. J. (2014). Adaptive parental effects: The importance of estimating environmental predictability and offspring fitness appropriately. Oikos, 123(7), 769–776. 10.1111/oik.01235

[ece37586-bib-0017] Chase, J. M. , & Leibold, M. A. (2003). Ecological niches: Linking classical and contemporary approaches. University of Chicago.

[ece37586-bib-0018] Chesson, P. (2000). Mechanisms and maintenance of species diversity. Annual Review of Ecology, Evolution, and Systematics, 31, 343–366. 10.1146/annurev.ecolsys.31.1.343

[ece37586-bib-0019] Chirgwin, E. , Marshall, D. J. , Sgrò, C. M. , & Monro, K. (2017). The other 96%: Can neglected sources of fitness variation offer new insights into adaptation to global change? Evolutionary Applications, 10(3), 267–275. 10.1111/eva.12447 28250811 PMC5322406

[ece37586-bib-0020] Dey, S. , Proulx, S. R. , & Teotónio, H. (2016). Adaptation to temporally fluctuating environments by the evolution of maternal effects. PLoS Biology, 14(2), e1002388. 10.1371/journal.pbio.1002388 26910440 PMC4766184

[ece37586-bib-0021] Duckworth, R. A. , Belloni, V. , & Anderson, S. R. (2015). Cycles of species replacement emerge from locally induced maternal effects on offspring behavior in a passerine bird. Science, 347(6224), 875–877. 10.1126/science.1260154 25700519

[ece37586-bib-0022] Dyer, A. R. , Brown, C. S. , Espeland, E. K. , McKay, J. K. , Meimberg, H. , & Rice, K. J. (2010). SYNTHESIS: The role of adaptive trans‐generational plasticity in biological invasions of plants. Evolutionary Applications, 3(2), 179–192. 10.1111/j.1752-4571.2010.00118.x 25567918 PMC3352481

[ece37586-bib-0023] Fox, C. W. , Czesak, M. E. , Mousseau, T. A. , & Roff, D. A. (1999). The evolutionary genetics of an adaptive maternal effect: Egg size plasticity in a seed beetle. Evolution; International Journal of Organic Evolution, 53(2), 552–560. 10.1111/j.1558-5646.1999.tb03790.x 28565419

[ece37586-bib-0024] Fox, C. W. , Thakar, M. S. , & Mousseau, T. A. (1997). Egg size plasticity in a seed beetle: An adaptive maternal effect. The American Naturalist, 149(1), 149–163. 10.1086/285983

[ece37586-bib-0025] Galloway, L. F. (2005). Maternal effects provide phenotypic adaptation to local environmental conditions. The New Phytologist, 166(1), 93–99. 10.1111/j.1469-8137.2004.01314.x 15760354

[ece37586-bib-0026] Galloway, L. F. , & Etterson, J. R. (2007). Transgenerational plasticity is adaptive in the wild. Science, 318(5853), 1134–1136. 10.1126/science.1148766 18006745

[ece37586-bib-0027] Geritz, S. A. H. (1995). Evolutionarily stable seed polymorphism and small‐scale spatial variation in seedling density. The American Naturalist, 146(5), 685–707. 10.1086/285820

[ece37586-bib-0028] Germain, R. M. , Caruso, C. M. , & Maherali, H. (2013). Mechanisms and consequences of water stress–induced parental effects in an invasive annual grass. International Journal of Plant Sciences, 174(6), 886–895. 10.1086/670691

[ece37586-bib-0029] Germain, R. M. , & Gilbert, B. (2014). Hidden responses to environmental variation: Maternal effects reveal species niche dimensions. Ecology Letters, 17(6), 662–669. 10.1111/ele.12267 24602193

[ece37586-bib-0030] Germain, R. , Grainger, T. , Jones, N. , & Gilbert, B. (2018). Maternal provisioning is structured by species' competitive neighborhoods. Oikos, 128(1), 45–53. 10.1111/oik.05530

[ece37586-bib-0031] Germain, R. M. , Weir, J. T. , & Gilbert, B. (2016). Species coexistence: Macroevolutionary relationships and the contingency of historical interactions. Proceedings of the Royal Society B: Biological Sciences, 283(1827), 20160047. 10.1098/rspb.2016.0047 PMC4822464 27009226

[ece37586-bib-0032] Ginzburg, L. R. , & Taneyhill, D. E. (1994). Population cycles of forest Lepidoptera: A maternal effect hypothesis. Journal of Animal Ecology, 63(1), 79–92. 10.2307/5585

[ece37586-bib-0033] Grafen, A. T. H. (1988). On the uses of data on lifetime reproductive success. University of Chicago Press.

[ece37586-bib-0034] Harrison, X. A. , Blount, J. D. , Inger, R. , Norris, D. R. , & Bearhop, S. (2011). Carry‐over effects as drivers of fitness differences in animals. The Journal of Animal Ecology, 80(1), 4–18. 10.1111/j.1365-2656.2010.01740.x 20726924

[ece37586-bib-0035] Herman, J. J. , & Sultan, S. E. (2011). Adaptive transgenerational plasticity in plants: Case studies, mechanisms, and implications for natural populations. Frontiers in Plant Science, 2, 1–10. 10.3389/fpls.2011.00102 22639624 PMC3355592

[ece37586-bib-0036] Herman, J. J. , Sultan, S. E. , Horgan‐Kobelski, T. , & Riggs, C. (2012). Adaptive transgenerational plasticity in an annual plant: Grandparental and parental drought stress enhance performance of seedlings in dry soil. Integrative and Comparative Biology, 52(1), 77–88. 10.1093/icb/ics041 22523124

[ece37586-bib-0037] Holeski, L. M. (2007). Within and between generation phenotypic plasticity in trichome density of *Mimulus guttatus* . Journal of Evolutionary Biology, 20(6), 2092–2100. 10.1111/j.1420-9101.2007.01434.x 17903186

[ece37586-bib-0038] Hoyle, R. B. , & Ezard, T. H. G. (2012). The benefits of maternal effects in novel and in stable environments. Journal of the Royal Society, Interface, 9(75), 2403–2413. 10.1098/rsif.2012.0183 PMC3427511 22572028

[ece37586-bib-0039] Jacob, S. , Bestion, E. , Legrand, D. , Clobert, J. , & Cote, J. (2015). Habitat matching and spatial heterogeneity of phenotypes: Implications for metapopulation and metacommunity functioning. Evolutionary Ecology, 29(6), 851–871. 10.1007/s10682-015-9776-5

[ece37586-bib-0040] Jakobsson, A. , & Eriksson, O. (2000). A comparative study of seed number, seed size, seedling size and recruitment in grassland plants. Oikos, 88(3), 494–502. 10.1034/j.1600-0706.2000.880304.x

[ece37586-bib-0041] Kawecki, T. J. , & Ebert, D. (2004). Conceptual issues in local adaptation. Ecology Letters, 7(12), 1225–1241. 10.1111/j.1461-0248.2004.00684.x

[ece37586-bib-0042] Larios, E. , & Venable, D. L. (2015). Maternal adjustment of offspring provisioning and the consequences for dispersal. Ecology, 96(10), 2771–2780. 10.1890/14-1565.1 26649397

[ece37586-bib-0043] Leibold, M. A. , & Chase, J. M. (2017). Metacommunity ecology (Vol. 59). Princeton University Press.

[ece37586-bib-0044] Levine, J. M. , & HilleRisLambers, J. (2009). The importance of niches for the maintenance of species diversity. Nature, 461(7261), 254–257. 10.1038/nature08251 19675568

[ece37586-bib-0045] Levine, J. M. , & Rees, M. (2004). Effects of temporal variability on rare plant persistence in annual systems. The American Naturalist, 164(3), 350–363. 10.1086/422859 15478090

[ece37586-bib-0046] Marshall, D. J. , & Burgess, S. C. (2015). Deconstructing environmental predictability: Seasonality, environmental colour and the biogeography of marine life histories. Ecology Letters, 18(2), 174–181. 10.1111/ele.12402 25534504

[ece37586-bib-0047] Marshall, D. J. , Monro, K. , Bode, M. , Keough, M. J. , & Swearer, S. (2010). Phenotype‐environment mismatches reduce connectivity in the sea. Ecology Letters, 13(1), 128–140. 10.1111/j.1461-0248.2009.01408.x 19968695

[ece37586-bib-0048] Marshall, D. J. , & Uller, T. (2007). When is a maternal effect adaptive? Oikos, 116, 1957–1963. 10.1111/j.2007.0030-1299.16203.x

[ece37586-bib-0049] McGinley, M. A. , Temme, D. H. , & Geber, M. A. (1987). Parental investment in offspring in variable environments: Theoretical and empirical considerations. The American Naturalist, 130(3), 370–398. 10.1086/284716

[ece37586-bib-0050] Monaghan, P. (2008). Early growth conditions, phenotypic development and environmental change. Philosophical Transactions of the Royal Society B: Biological Sciences, 363(1497), 1635–1645. 10.1098/rstb.2007.0011 PMC2606729 18048301

[ece37586-bib-0051] Moore, M. P. , Whiteman, H. H. , & Martin, R. A. (2019). A mother's legacy: The strength of maternal effects in animal populations. Ecology Letters, 22(10), 1620–1628. 10.1111/ele.13351 31353805

[ece37586-bib-0052] Mousseau, T. A. , & Dingle, H. (1991). Maternal effects in insect life histories. Annual Review of Entomology, 36(1), 511–534. 10.1146/annurev.en.36.010191.002455

[ece37586-bib-0053] Mousseau, T. A. , & Fox, C. W. (1998). The adaptive significance of maternal effects. Trends in Ecology & Evolution, 13(10), 403–407. 10.1016/S0169-5347(98)01472-4 21238360

[ece37586-bib-0054] Mousseau, T. A. , Uller, T. , Wapstra, E. , & Badyaev, A. V. (2009). Evolution of maternal effects: Past and present. Philosophical Transactions of the Royal Society B: Biological Sciences, 364(1520), 1035–1038. 10.1098/rstb.2008.0303 PMC2666690 19324608

[ece37586-bib-0055] Ogle, K. , Barber, J. J. , Barron‐Gafford, G. A. , Bentley, L. P. , Young, J. M. , Huxman, T. E. , Loik, M. E. , & Tissue, D. T. (2015). Quantifying ecological memory in plant and ecosystem processes. Ecology Letters, 18(3), 221–235. 10.1111/ele.12399 25522778

[ece37586-bib-0056] Potticary, A. L. , & Duckworth, R. A. (2020). Multiple environmental stressors induce an adaptive maternal effect. The American Naturalist, 196(4), 487–500. 10.1086/710210 32970461

[ece37586-bib-0057] Räsänen, K. , & Kruuk, L. E. B. (2007). Maternal effects and evolution at ecological time‐scales. Functional Ecology, 21(3), 408–421. 10.1111/j.1365-2435.2007.01246.x

[ece37586-bib-0058] Rees, M. , & Westoby, M. (1997). Game‐theoretical evolution of seed mass in multi‐species ecological models. Oikos, 78(1), 116–126. 10.2307/3545807

[ece37586-bib-0059] Roach, D. A. , & Wulff, R. D. (1987). Maternal effects in plants. Annual Review of Ecology and Systematics, 18(1), 209–235. 10.1146/annurev.es.18.110187.001233

[ece37586-bib-0060] Rossiter, M. (1996). Incidence and consequences of inherited environmental effects. Annual Review of Ecology and Systematics, 27(1), 451–476. 10.1146/annurev.ecolsys.27.1.451

[ece37586-bib-0061] Smith, C. C. , & Fretwell, S. D. (1974). The optimal balance between size and number of offspring. The American Naturalist, 108(962), 499–506. 10.1086/282929

[ece37586-bib-0062] Song, Z. , Zou, Y. , Hu, C. , Ye, Y. , Wang, C. , Qing, B. , Komdeur, J. , & Ding, C. (2018). Silver spoon effects of hatching order in an asynchronous hatching bird. Behavioral Ecology, 30(2), 509–517. 10.1093/beheco/ary191

[ece37586-bib-0063] Stearns, S. C. (1992). The evolution of life histories. Oxford University Press.

[ece37586-bib-0064] Stratton, D. A. (1989). Competition prolongs expression of maternal effects in seedlings of *Erigeron annuus* (Asteraceae). American Journal of Botany, 76(11), 1646–1653. 10.1002/j.1537-2197.1989.tb15149.x

[ece37586-bib-0065] Sultan, S. E. , Barton, K. , & Wilczek, A. M. (2009). Contrasting patterns of transgenerational plasticity in ecologically distinct congeners. Ecology, 90(7), 1831–1839. 10.1890/08-1064.1 19694132

[ece37586-bib-0066] Tilman, D. , Mattson, M. , & Langer, S. (1981). Competition and nutrient kinetics along a temperature gradient: An experimental test of a mechanistic approach to niche theory 1. Limnology and Oceanography, 26(6), 1020–1033. 10.4319/lo.1981.26.6.1020

[ece37586-bib-0067] Tollrian, R. (1995). Predator‐induced morphological defenses: Costs, life history shifts, and maternal effects in *Daphnia pulex* . Ecology, 76(6), 1691–1705. 10.2307/1940703

[ece37586-bib-0068] Van Allen, B. G. , & Rudolf, V. H. W. (2013). Ghosts of habitats past: Environmental carry‐over effects drive population dynamics in novel habitat. The American Naturalist, 181(5), 596–608. 10.1086/670127 23594544

[ece37586-bib-0069] Van Allen, B. G. , & Rudolf, V. H. W. (2015). Habitat‐mediated carry‐over effects lead to context‐dependent outcomes of species interactions. The Journal of Animal Ecology, 84(6), 1646–1656. 10.1111/1365-2656.12408 26060938

[ece37586-bib-0070] Van Allen, B. G. , & Rudolf, V. H. W. (2016). Carryover effects drive competitive dominance in spatially structured environments. Proceedings of the National Academy of Sciences of the United States of America, 113(25), 6939–6944. 10.1073/pnas.1520536113 27298356 PMC4922188

[ece37586-bib-0071] Van de Pol, M. , Bruinzeel, L. W. , Heg, D. , Van der Jeugd, H. P. , & Verhulst, S. (2006). A silver spoon for a golden future: Long‐term effects of natal origin on fitness prospects of oystercatchers (*Haematopus ostralegus*). The Journal of Animal Ecology, 75(2), 616–626. 10.1111/j.1365-2656.2006.01079.x 16638014

[ece37586-bib-0072] Vance, R. R. (1973). On reproductive strategies in marine benthic invertebrates. The American Naturalist, 107(955), 339–352. 10.1086/282838 18811275

[ece37586-bib-0073] Vasilieva, N. A. , & Tchabovsky, A. V. (2020). Early predictors of female lifetime reproductive success in a solitary hibernator: Evidence for “silver spoon” effect. Oecologia, 193(1), 77–87. 10.1007/s00442-020-04649-1 32318852

[ece37586-bib-0074] Vasseur, D. A. , & Yodzis, P. (2004). The color of environmental noise. Ecology, 85(4), 1146–1152. 10.1890/02-3122

[ece37586-bib-0075] Yuan, C. , & Chesson, P. (2015). The relative importance of relative nonlinearity and the storage effect in the lottery model. Theoretical Population Biology, 105, 39–52. 10.1016/j.tpb.2015.08.001 26307205

