# Supplementary material for: Maternal effects and the outcome of interspecific competition
Source: Ecol Evol. 2021 May 2;11(12):7544–56. doi: 10.1002/ece3.7586 (PMC8216948; doi:10.1002/ece3.7586)
Supplement: Supplementary file 1 — Supplementary Material [file ECE3-11-7544-s001.docx]

**Supplementary materials**

**(a) Supplementary figures and tables**


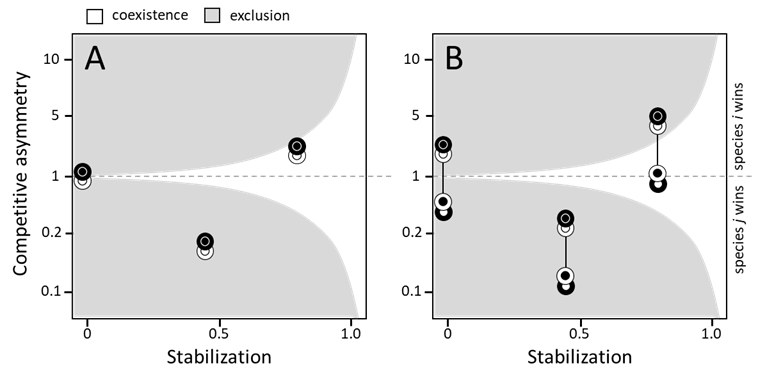


**Figure S1.** Conceptual example of how the impacts of maternal effects on competitive dynamics are general despite different pre-existing degrees of stabilization vs. competitive asymmetry. Points show maternal conditions (outer circle) and offspring conditions (inner circle)—when outer and inner colors are the same, maternal and offspring conditions match. Overlapping points are jittered vertically. (A) In the absence of maternal effects, outcomes are insensitive to maternal environmental conditions, whereas (B) maternal conditions strongly influence the magnitude and direction of competitive asymmetry in the presence of maternal effects. Neutral interactions occur when stabilization is absent and competitive asymmetries are 1, whereas non-neutral interactions are any divergence from these criteria.


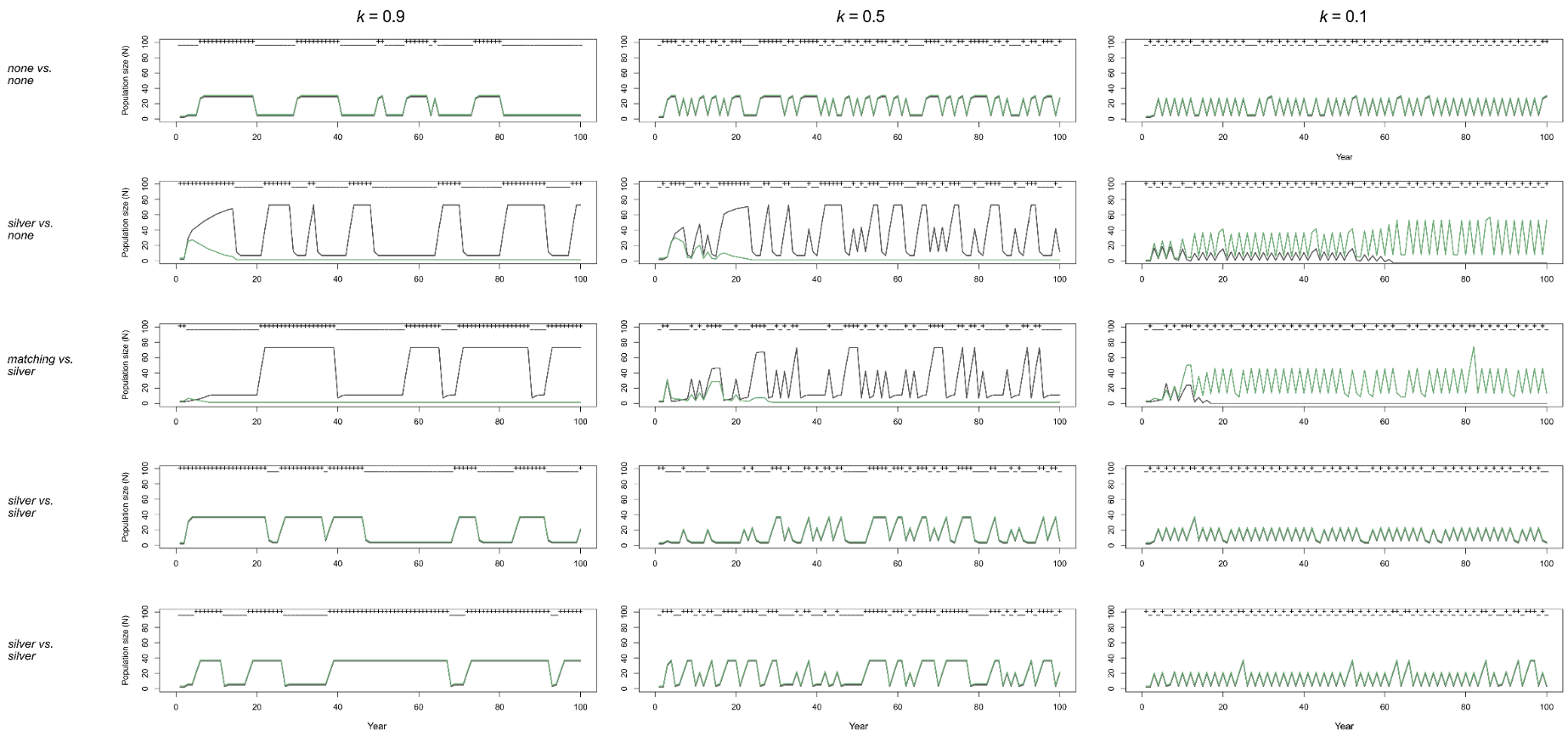


**Figure S2.** Competitive dynamics of pairs of species (species *i* ( grey), species *j* (green)) that differ in maternal effects phenotype, shown in the left margin. Pairs in which both species have the same phenotype co-occur neutrally indefinitely and exhibit the same dynamics, thus the grey and black lines overlap. We show dynamics at high (0.9), intermediate (0.5), and low (0.1) levels of temporal autocorrelation (*k*). Environmental conditions in each year, good (+) and bad (-), are shown at top of each panel, and only the first 100 years of each simulation are shown. Note that due to the probabilistic sampling of environmental conditions, each replicate simulation produces a different environmental structure and thus different dynamics. Summaries of dynamical outcomes across 500 replicate simulations per phenotype combination are presented in figure 3.


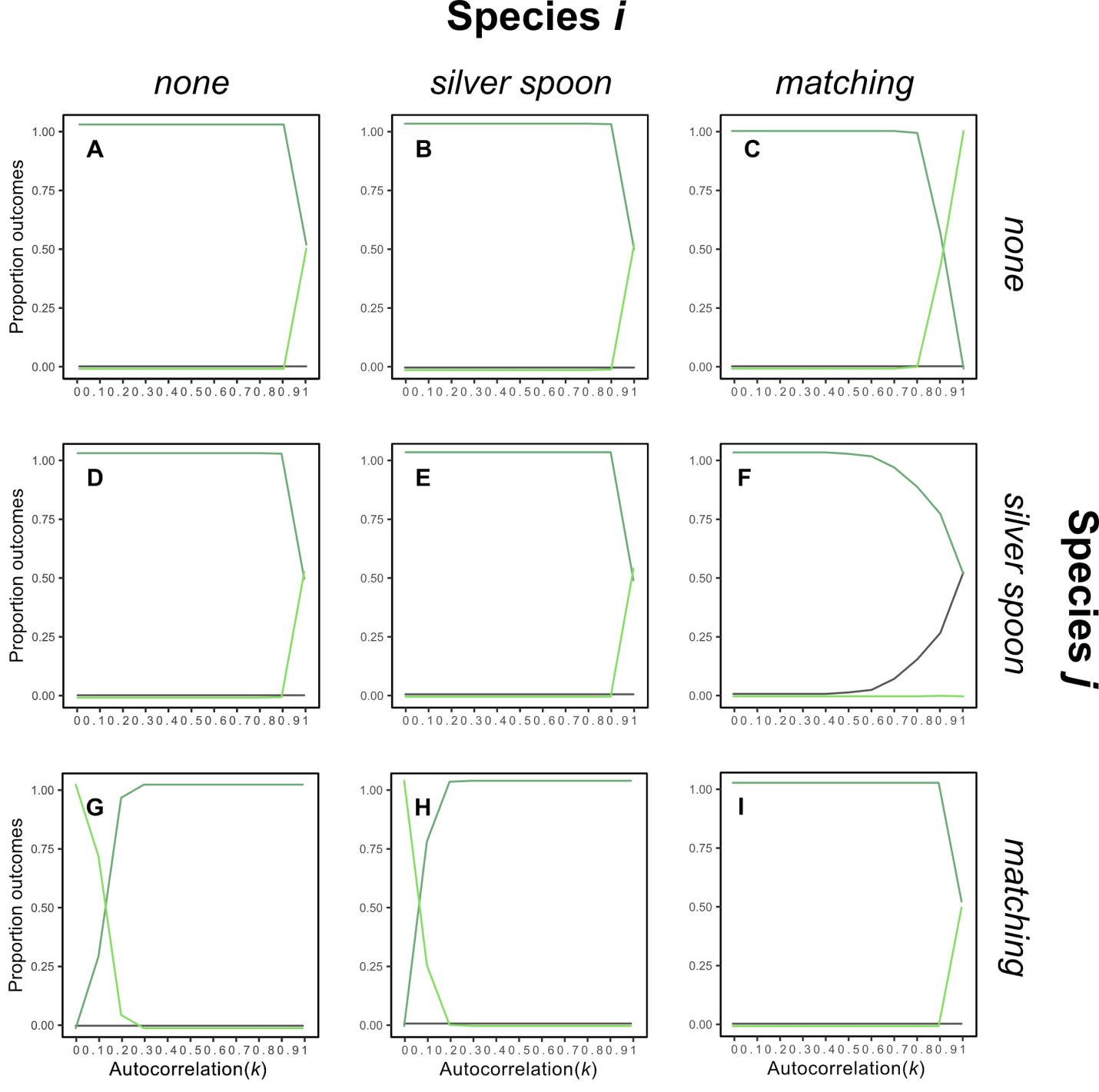


**Figure S3.**

Proportion of simulation outcomes in which only species *i* (dark grey line), only species *j* (dark green line), or both species (light green line) are present at the end of the 500-year simulation, depending on the type of maternal effects exhibited by each species and the level of temporal autocorrelation (*k*). Autocorrelation of 1.0 corresponds to constant conditions, 0.5 is a 50% chance of switching conditions among years, and 0.0 is 100% chance of switching (see figure 2 for examples of how changing k affects the temporal sequence of environmental conditions). Competition coefficients are *α_ii_* = 1.25, *α_jj_* = 1.0, *α_ij_* = 1.0, and *α_ji_* = 0.8. The dynamics of two scenarios (panels C,G) are expanded in figure 5.

**Table S1.** How intrinsic rates of increase (*λ*_(_*_i,o_*_)_; eq. (1)) are adjusted by maternal environmental effects and seed size-number tradeoffs, as per eq. (2).

| **Phenotype** | **Quality of environment** | | ***λ*_(_*_i,o_*_)_** | ***M*** | ***s^M^*** | ***λ_i_*(*t*)** |
| --- | --- | --- | --- | --- | --- | --- |
|  | ***t-1 (m)*** | ***t (o)*** |  |  |  |  |
| none | + | + | 60 | 0 | 1 | 60 |
|  | + | - | 10 | 0 | 1 | 10 |
|  | - | - | 10 | 0 | 1 | 10 |
|  | - | + | 60 | 0 | 1 | 60 |
| silver spoon | + | + | 60 | -1 | 0.8 | 75 |
|  | + | - | 10 | -1 | 0.8 | 10 |
|  | - | - | 10 | -1 | 1.25 | 8 |
|  | - | + | 60 | -1 | 1.25 | 48 |
| matching | + | + | 60 | -1 | 0.8 | 75 |
|  | + | - | 10 | 1 | 0.8 | 8 |
|  | - | - | 10 | 1 | 1.25 | 12.5 |
|  | - | + | 60 | -1 | 1.25 | 48 |
